# Hand-foot Syndrome Secondary to Low-dose Docetaxel in a Breast Cancer Patient

**DOI:** 10.7759/cureus.4400

**Published:** 2019-04-06

**Authors:** Tariq Kewan, Mohammad Alomari, Shrouq Khazaaleh, Fahrettin Covut, May Olayan

**Affiliations:** 1 Internal Medicine, Cleveland Clinic - Fairview Hospital, Cleveland, USA; 2 Internal Medicine, Cleveland Clinic Foundation, Cleveland, USA

**Keywords:** hand-foot syndrome, docetaxel

## Abstract

Docetaxel-induced hand-foot syndrome (HFS) at low doses is a very rare side effect that usually occurs in a dose-dependent manner. HFS can be managed with conservative measures and may need chemotherapy discontinuation. In this report we present a case of HFS in a breast cancer patient after one dose of docetaxel.

## Introduction

Hand-foot syndrome (HFS), also known as palmoplantar erythrodysesthesia or Burgdorf’s reaction, is a localized cutaneous reaction associated with the administration of several chemotherapeutic agents. Palms and soles erythema, swelling, and paresthesia are the most common presenting symptoms. The most common agents causing HFS are doxorubicin, 5-fluorouracil, capecitabine, vinorelbine, and methotrexate. HFS induced by docetaxel is a rare, dose-dependent adverse reaction [1-3].

## Case presentation

**Figure 1:**
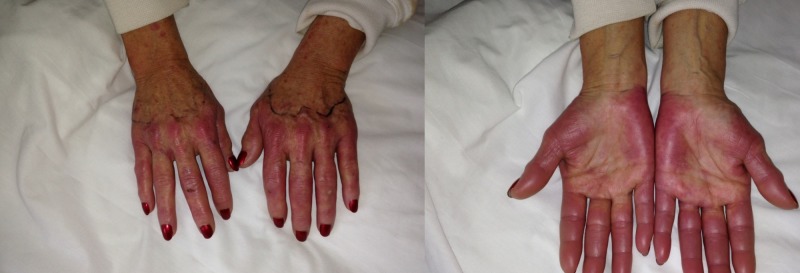
Dorsal and plantar view of both hands showing erythema and swelling bilaterally.

A 51-year-old female patient with a past medical history of stage IIIa (T1c, N2a, M0) right breast cancer, hepatitis C infection, and hypertension presented to emergency department (ED) with bilateral hand swelling, redness, and edema that started three days before coming to ED. Swelling and redness started only one day after starting docetaxel chemotherapy. She received a single dose of 117 mg (75 mg/m^2^) intravenous docetaxel. Also, she received prednisone before starting chemotherapy. She denied any recent fever or chills. Vitals signs were stable on admission; no fever was documented. Physical examination revealed bilateral swelling, redness, and tenderness of both hands up to the wrists (Figure 1). No upper limb weakness was found on physical examination. No skin rash was observed in other body parts.

Lab investigation showed normal white blood cells count of 7.24 thousand cell/ul (normal range: 3.70-11.00 k/uL). Sepsis lactate was checked and was found to be 1.3 mmol/L (normal range: 0.5-2.0 mmol/L). Basal metabolic panel, c-reactive protein (CRP), and erythrocyte sedimentation rate (ESR) on admission were unremarkable. Blood cultures were done and did not grow any microorganism. No imaging studies were done.

The patient was given vancomycin for one day without any improvement in skin rash or hand edema. On the next day antibiotics was stopped by the infectious disease team. She was then started on intravenous 40 mg methylprednisolone. Swelling, redness, and pain started to improve 24 h after steroid initiation. The patient was discharged on prednisone 20 mg three times daily for another seven days. She was diagnosed with HFS erythrodysesthesia.

## Discussion

Hand-foot syndrome is a distinctive cutaneous toxicity affecting the palms and/or soles after exposure to certain chemotherapeutic agents. While most cases are induced by capecitabine, 5-fluorouracil, and doxorubicin, with a reported incidence up to 50%, docetaxel-induced HFS is considered rare and dose-dependent, with only few cases reported to date [4-7]. Typically, it manifests with numbness, itching sensation, redness, and swelling of limbs [8].

Docetaxel is used in the management of locally advanced or metastatic breast carcinoma. The main side effect of docetaxel is bone marrow suppression; it can also lead to skin toxicity in a dose-dependent manner [9-10]. Few cases of low dose (75 mg/m^2^) docetaxel-induced HFS have been reported. In one case report, a 45-year-old female patient with breast cancer was reported to have docetaxel-induced HFS after receiving low dose (75 mg/m^2^) docetaxel [11]. Another case of docetaxel-induced HFS was reported in a 62-year-old female patient with invasive ductal breast cancer after receiving a similar dose [1].

Pathogenesis of docetaxel-induced HFS remains largely unknown. Rapidly dividing epidermal basal cells in the palms and soles are most sensitive to cytotoxic effects of docetaxel; making these areas more vulnerable for HFS. Other factors like temperature, pressure, and friction movements of hands and feet can increase the risk to HFS [12]. Docetaxel is metabolized by liver cytochrome P450 3A4 (CYP3A4) enzyme; inhibition of this enzyme decreases docetaxel metabolism and increases the risk for HFS [11-12]. In the present report, no other risk factors for HFS were identified.

Docetaxel-induced HFS is a clinical diagnosis. Skin biopsies can be used in some cases to differentiate it from cellulitis or other differential diagnoses. Our patient presented with a bilateral skin involvement making cellulitis diagnosis less likely [13]. Treatment of HFS is usually achieved by medication discontinuation and conservative measures such as ice bags, avoidance of sun exposure, and topical emollients. In severe cases, docetaxel can be stopped or restarted at lower doses if applicable [1, 3, 14]. Corticosteroids and pyridoxine have been found to be effective in HFS treatment and prophylaxis in some studies [15-16]. Our patient was managed by conservative measures and received oral steroids as well.

## Conclusions

Low-dose docetaxel-induced HFS is very rare, and usually occurs in a dose-dependent manner. Although not serious, management of HFS improves the quality of life among active cancer patients. It usually responds to medication discontinuation and conservative measures. Physicians should be aware of this adverse event and the necessity to stop or at least decrease the dose of the offending agent expecting a complete resolution of symptoms afterwards.
